# Capacitance Contribution of NIH/3T3 Cells Existing on and between Electrodes of an Impedance Biosensor

**DOI:** 10.3390/bios13110970

**Published:** 2023-11-06

**Authors:** Yeeun Kim, Dahyun Kang, Seokgyu Kim, Eunchae Hong, Moongyu Jang

**Affiliations:** 1 School of Semiconductor & Display Technology, Hallym University, Chuncheon 24252, Republic of Korea; h20175407@hallym.ac.kr (Y.K.); h20185401@hallym.ac.kr (D.K.); hakillike98@hallym.ac.kr (S.K.); 2 Department of Life Science, Hallym University, Chuncheon 24252, Republic of Korea; giok9901@gmail.com; 3 Center of Nano Convergence Technology, Hallym University, Chuncheon 24252, Republic of Korea

**Keywords:** impedance biosensor, NIH 3T3 cell, capacitance

## Abstract

In this study, an impedance biosensor capable of real-time monitoring of the growth and drug reactions using NIH/3T3 cells was fabricated through a semiconductor process. With the fabricated impedance biosensor, the cell growth and drug reaction states are monitored in real-time, showing the validness of the developed biosensor. By using the developed impedance biosensor, we have investigated the capacitance contribution of NIH/3T3 cells existing on electrodes and between electrodes. To compare the capacitance value contributions of the cells on and between electrodes, wide- and narrow-gap electrode patterns are manufactured with 3.7 and 0.3 mm electrode gap spacings, respectively. From the detailed analysis, the capacitance contributions of NIH/3T3 cells existing on electrodes are estimated around less than 20 percent compared to the cells existing between electrodes. In other words, a minimized electrode area with maximized electrode spacing is the promising impedance biosensor design guide for accurate cell capacitance measurements.

## 1. Introduction

Biosensor research is rapidly advancing due to increased interest in healthcare and disease diagnoses [1,2]. The needs for biosensors are expanding in various fields such as disease diagnosis kits, healthcare wearable devices, and the detection of food poisoning bacteria [3,4]. For the applications of biosensors, selectivity, reproducibility, stability, and sensitivity are the key factors that determine their performances [5]. Also, there are high demands for improvements in the performances of biosensors, and many efforts are being made to achieve this goal [6,7]. For instance, in the cases of biosensors used for determining COVID-19 infection, combining them with nanotechnology has increased their reliability and selectivity, enabling quick and accurate diagnosis [8]. As such, numerous studies on next-generation biosensors are underway to improve their performances and analyses [9,10].

Recently, as an electrical method, impedance biosensors are being frequently studied as one of the representative techniques due to the highly sensitive measurement of various cell activities [11]. One representative electrical measurement method for impedance biosensors is the analysis method called ECIS (Electric Cell-substrate Impedance Sensing), which measures impedance by applying an electric field to cells cultured on an electrode. Giaever and Keese first introduced this technology in the late 20th century [12]. ECIS is a non-destructive and real-time technology that is used to identify changes in various reactions, such as specific proteins, cell receptors, and the number of cells, and monitor them with electrical signals [13]. In impedance measurements, a small amplitude AC voltage is applied to the sensor electrode to measure the change in gap and phase as a function of frequency [14]. With the introduction of the ECIS technique, real-time monitoring is possible, and they have considerable potential as simple and portable sensors [15].

Recent research has predominantly focused on impedance biosensors that utilize the ECIS technique. A team at Zhejiang University introduced a novel multi-functional biosensor equipped with both a microelectrode (ME) and an interdigital electrode (IDE). Leveraging the ECIS technique, this biosensor assesses the effects of non-small cell lung cancer inhibiting drugs and their cardiac toxicity. One particular study demonstrated that the drug AC0010 effectively targeted epidermal growth factor receptor mutations in non-small-cell lung cancer patients by measuring impedance changes. This study also evaluated the drug’s influence on myocardial cell function [16]. In a separate line of research, a German team examined the role of electrical stimulation in bone formation during fracture healing, laying the groundwork for a potential therapeutic device. A sensor equipped with a gold electrode was fabricated, employing the ECIS technique to detect impedance variations in seeded cells. This method elucidated the differentiation of stem cells via electrical stimulation [17]. Separately, researchers from the Department of Convergence Engineering at Chung-Ang University developed a gold microelectrode specifically for analyzing human gastric cancer cells (MKN-1) using ECIS. Their approach involved depositing a composite film made of reduced graphene oxide (RGO) and polyaniline (PANI) to effectively conduct cell growth analysis. Furthermore, they monitored the growth of MKN-1 cells and assessed the efficacy of curcumin, a known anti-cancer drug [18]. The research team at the National Taiwan University of Science and Technology has developed a bioelectrode based on a conducting polymer, poly(3,4-ethylenedioxythiophene)-poly-(styrenesulfonate) (PE-DOT: PSS), enhancing ECIS technology. This enabled efficient monitoring of the cytotoxic response of anti-cancer drugs on HeLa and MCF7 cells [19]. Separately, the Department of Life Sciences at Northwest University of Science and Technology introduced an ECIS sensor array equipped with various electrodes designed to observe MC3T3-E1 cells, which are similar to osteoblasts. Objective measurements indicated that impedance detection varies depending on electrode diameter and number, providing foundational insights for electrode design. Specifically, electrodes 25 µm or smaller showcased the potential for single-cell detection [20]. Given these advancements, there is ongoing extensive research aimed at enhancing the versatility and viability of the ECIS method for a plethora of applications.

In this study, an impedance biosensor is fabricated through semiconductor process for the real-time monitoring of the growth and drug reaction of NIH/3T3 cells. Using the impedance biosensor, the NIH/3T3 cell growth and drug reaction states are monitored in real-time, showing the validness of the developed biosensor. In particular, it is difficult to find studies that address how the performances of impedance biosensors depend on their design patterns. Therefore, using the developed impedance biosensor, we have investigated the capacitance contribution of NIH/3T3 cells existing on electrode and between electrodes. For the comparison, wide- and narrow-gap electrode patterns are designed with 3.7 and 0.3 mm electrode gap spacings, respectively. By using a parallel connection model between the cells existing on and between electrodes, we have established a numerical model. From the model and the measured capacitance values, we have extracted the cell capacitance components by the cells existing on and between sensor electrodes. Based on our results, we will propose an optimized design guideline to enhance the sensitivity of cell impedance measurements. The detailed methods and analysis will be given in the following.

## 2. Materials and Methods

### 2.1. Impedance Biosensor Fabrication Process

**Figure 1:**
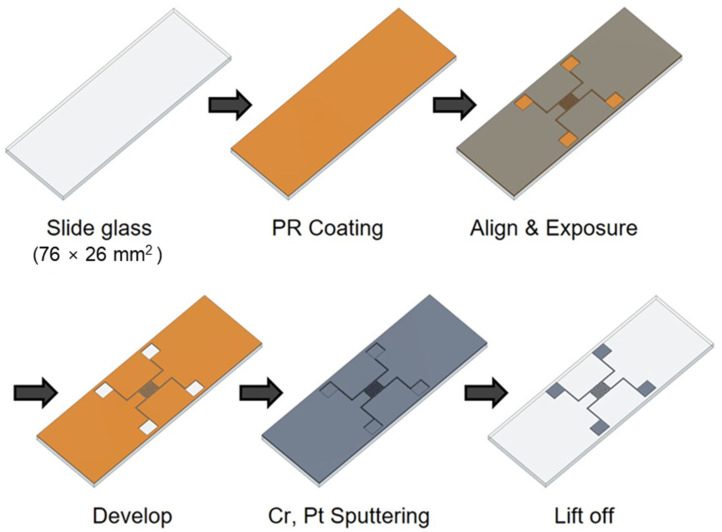
Manufacturing process of impedance biosensor using semiconductor processes.

The impedance biosensor was fabricated using a semiconductor process, as shown in Figure 1. The biosensor was manufactured on a glass slide to enable easy observation of cell growth and drug response. To create a pattern, positive PR (photoresist, AZ 5214) was spin coated at 4500 rpm for 35 s using a spin coater. The PR-coated glass slide was then baked at 100 °C for 10 min to improve the adhesion between the PR and the glass slide. A shadow mask was aligned on the glass to produce impedance biosensor patterns, respectively. Wide and narrow patterns had electrode widths of 0.3 and 2 mm, respectively, and electrode spacings of 3.7 and 0.3 mm, respectively. The shadow mask was exposed to ultraviolet (UV) radiation for 8.3 s using a contact aligner (EVG 610). The pattern was developed by soaking the samples in a developer (AZ 300 MIF Developer) for 1 min and confirming the removal PR with a microscope. Metal was deposited on the pattern using an RF/DC magnetron sputter (SHS-2M3-40T). First, chromium (Cr) was deposited to improve the adhesion between platinum (Pt) and the glass, and Pt was deposited as the electrode metal due to its excellent biocompatibility. The deposited thicknesses of Cr and Pt were 2 and 12 nm, respectively. After Cr and Pt deposition, the lift-off technique was used for the removal of remained PR in an ultrasonic cleaner for 3 min. Then, a thermal annealing process (Furnace, SHTC-3000) was performed for 1 h using Argon (Ar) gas at 400 °C. For the stable electrical contact, 2000 Å thick aluminum (Al) was deposited on the pad part using a thermal evaporator (SHE-6T-350D) with the shadow mask method. The shadow mask was manufactured by Yesung (Ansan, Republic of Korea). Additionally, all the materials used in this study were purchased from Medicoapex (Chuncheon, Republic of Korea) and 4 science (Seongnam, Republic of Korea). Finally, the impedance biosensor pad parts were connected using wires to multi-connectors for the electrical signal monitoring. The next steps for the cell seeding process are explained in the following section.

### 2.2. NIH/3T3 Cell Growth and Drug Reaction Process 

**Figure 2:**
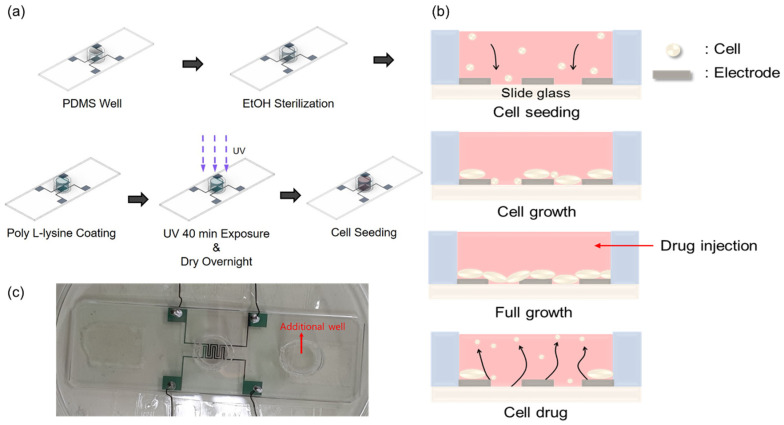
Cell seeding procedures. (**a**) Well internal environment creation process. (**b**) Growth process and drug responses of cells inside the well. (**c**) Real impedance biosensor (all used wires are insulated for electrical separation). The PDMS well dimensions are 8, 10, and 7 mm for the inner and outer diameters and heights, respectively. In (**c**), an additional well is introduced to minimize the volume change in medium in the main PDMS well during the measurements.

To study the growth and drug response characteristics of cells on the impedance biosensor, a suitable environment was created, as shown in Figure 2a. A biopsy punch was used to create a PDMS (poly-dimethylsiloxane) well with an inner diameter of 8 mm, an outer diameter of 10 mm, and a capacity of 350 μL. A mixture of PDMS and hardener in a ratio of 5:1 was used as the adhesive. This adhesive was applied to the bottom of the well, surrounding the pattern. At this time, one well was attached to the center of the pattern for the purpose of seeding cells, and one additional well was attached to the outside of the pattern to maintain humidity by injecting DPBS. The attached wells were dried on a hot plate at 100 °C for 2 h. For the confirmation of stabled and reproducible attachment of the PDMS well, we performed microscope inspection and a measurement of resistance and capacitance values. Although the patterns had sufficient spacing margins, misplacement could be checked easily by microscope inspection. Moreover, before the cell seeding, we checked resistance and capacitance values in dry conditions for the reliability of the data obtained during the cell experiments. Prior to cell seeding, the interior of the well was sterilized to avoid contamination by first rinsing the well with 350 μL PBS (phosphate-buffered saline). This was followed by a wash with 350 μL of 70% EtOH, which was allowed to stand for 1 min before being removed. This rinsing process was repeated three times to ensure sterilization. To improve cell adhesion on the surface of the glass slide, the well was treated with 350 μL of poly-L-lysine 0.01 wt%. In all steps, the precise 350 μL volume was controlled by using an Eppendorf research 100–1000 μL volumetric adjustable pipette. Coating was carried out for 40 min, during which time UV irradiation was applied for sterilization. The well was then left to dry overnight and prepared for cell growth. Finally, 10,000 cells were seeded into the well. 

Figure 2b illustrates the cell growth process and drug response observed 48 h after cell seeding in the well. After seeding, the initially floating cells settle on the glass slide due to gravitational forces. These cells then continue to divide and proliferate until the slide surface is saturated. Once saturation of cell division and growth is reached, puromycin (Gibco™, New York, NY, USA, A11138-03) is injected at a concentration of 5.72 μg/mL to induce cell death.

Figure 2c shows the actual impedance biosensor used in this study. By fabricating an impedance biosensor using this process, and measuring signals using the ECIS analysis method, cell behavior can be conveniently obtained as a change in capacitance. It is also more meaningful because all cell changes that occur within the well can be represented as a single data point. 

### 2.3. NIH/3T3 Cell Culture

The cells used in this study were NIH/3T3 mouse embryonic cells in the form of fibroblasts. NIH/3T3 cells have a size of about 18 μm immediately after dispensing, and, when they attach to a surface and grow, the cell size increases, and the surface area expands, reaching a size of about 100 μm or more [21,22]. The medium used for cell culture was CORNING’s Cat. NO. 10-013-CV DMEM (Dulbecco’s Modification of Eagle’s Medium), 1× with 4.5 g/L glucose, L-glutamine, and sodium pyruvate. To this medium, 10% calf serum and 1% streptomycin were added as antibiotics. To induce cell death, we used puromycin at a base concentration of 10 mg/mL, which was subsequently diluted in the medium to 10 μg/mL. Throughout the culture process, the CO_2_ incubator (Thermo Electron Corporation, Waltham, MA, USA) was maintained at 37 °C, with humidity maintained above 95% and CO_2_ concentration at 5%. 

### 2.4. Impedance Biosensor Design: Reference, Wide, and Narrow Patterns

**Figure 3:**
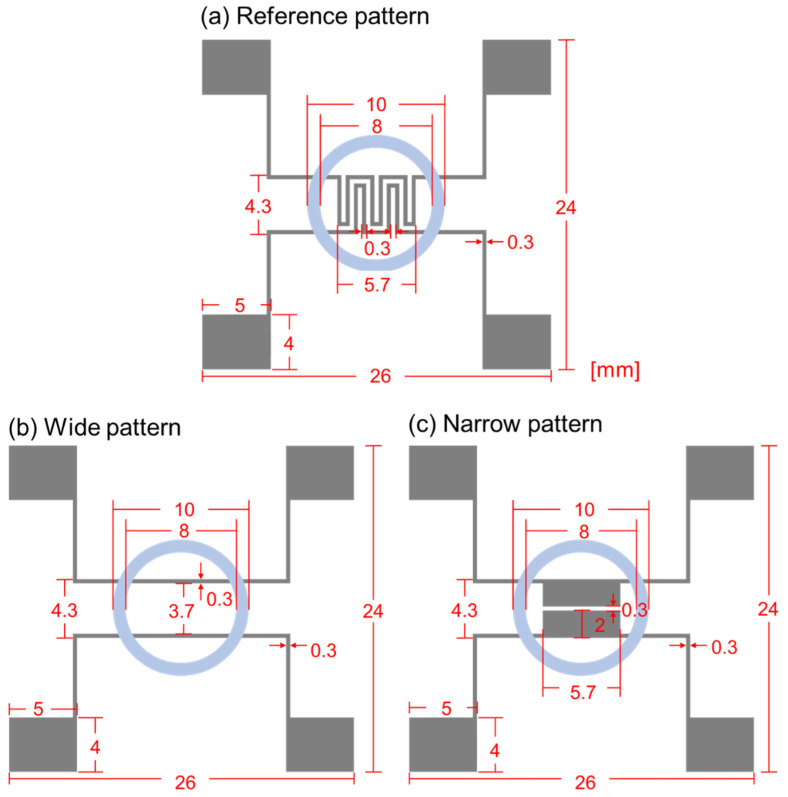
Schematic diagram of impedance biosensors for (**a**) reference pattern, (**b**) wide pattern, and (**c**) narrow pattern.

Figure 3 shows the schematic diagram of the biosensor’s designs, and the specific dimensions are specified in mm scale for the clearness. Figure 3a–c represent reference, wide, and narrow patterns, respectively. In the reference pattern, the electrode width and spacing are both 0.3 mm, and, for the wide pattern, the electrode width is also 0.3 mm, but the spacing is set as 3.7 mm. The reference pattern was frequently used in our group for the cell growth and cell–drug reaction monitoring, and 0.3 mm width and spacing were chosen due to the pattern formation stability during lift-off process. Through all three patterns, PDMS well sizes were set as the same, and the inner and outer diameters were 8 and 10 mm, respectively. In the narrow pattern, the electrode width was 2 mm. and the spacing was 0.3 mm. Thus, compared to the reference pattern, the wide pattern had enlarged electrode spacing, and the narrow pattern had enlarged electrode width. Except for the design variations within the PDMS well, all the other parts’ designs, including the pads, were the same.

### 2.5. Electrical Measurement Methods

**Figure 4:**
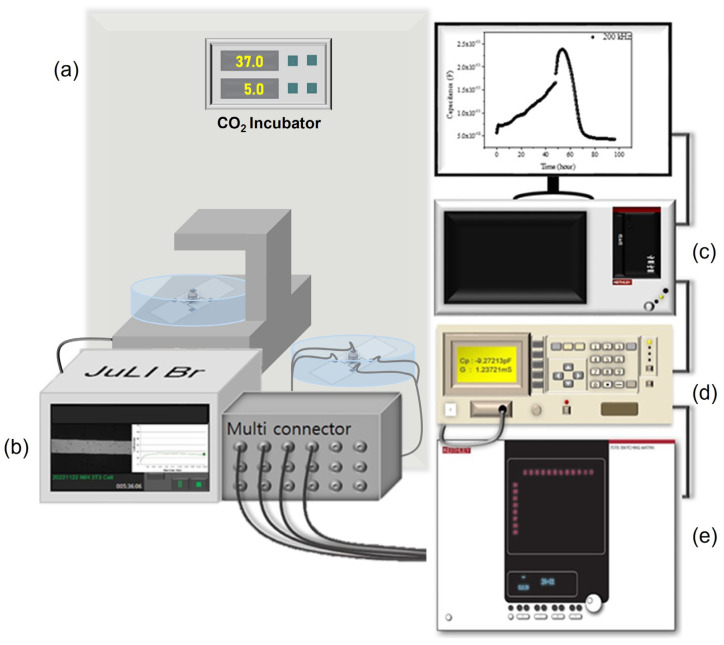
(**a**) CO_2_ incubator, (**b**) JuLi^TM^ Br&FL, (**c**) Keithley 4200-SCS, (**d**) Agilent 4284A, and (**e**) Keithley 707B switching matrix denotes experimental set-up.

Figure 4 shows the electrical measurement schematic for the impedance signal measurement on the impedance biosensor. Keithley 4200-SCS controlled the whole system, and Agilent 4284A (20 Hz to 1 MHz) measured the impedance values with frequency. For the multi-sensor signal detection, a Keithley 707B switching matrix was used using a multi-connector. For the biological status monitoring, microscopic images were obtained using NanoEnTek’s live cell movie analyzer (JuLi^TM^ Br&FL, Waltham, MA, USA). Capacitance–frequency data were monitored every minute for 96 h, with frequency changes ranging from 1 kHz to 1 MHz using the aforementioned electrical measurement set up. 

The integration time was set to mid (short, mid, and long) range. The excitation amplitude was set to 100 mV. Of course, we measured capacitance and the phase (usually 4284A measures the real and imaginary parts of impedance) and tried to analyze the phase part. However, it was hard to analyze the phase part, and most of the research groups used capacitance rather than phase signal due to the same reason.

## 3. Results and Discussion

### 3.1. Capacitance Monitoring of NIH/3T3 Cell Using 0.3 mm Reference Pattern

**Figure 5:**
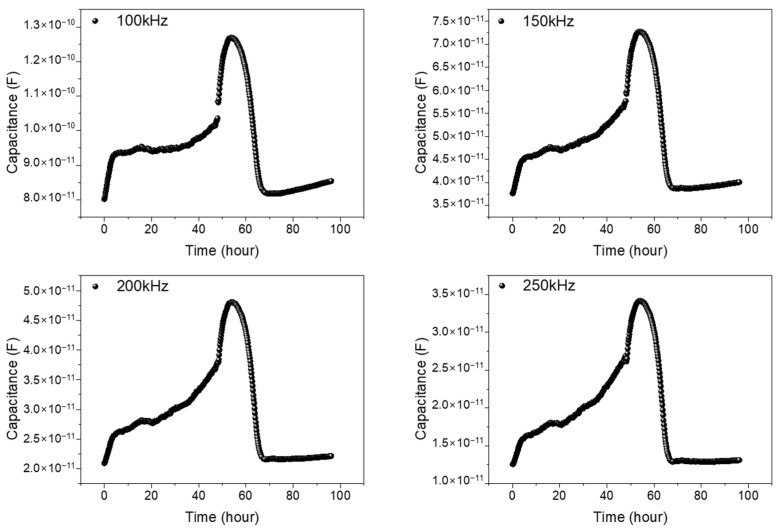
Capacitance variations vs. time of NIH/3T3 cell in 0.3 mm reference pattern. The monitored frequencies are 100, 150, 200, and 250 kHz. After NIH/3T3 cell seeding (0 h), the measured capacitance values show a gradual increase up to 96 h due to the cell growth. At 96 h, Cisplatin is injected, and the capacitance rapidly decreases due to the cell–drug reactions.

First, for the validation of the impedance measurement technique, the 0.3 mm reference pattern was used for the monitoring of the NIH/3T3 cell growth and cell–drug reactions using Puromycin. Puromycin is frequently used to kill cells, and it has a type of antibiotic that functions as a protein synthesis inhibitor. In the 0.3 mm reference pattern, a stable signal could be obtained in the frequency range between 100 and 250 kHz. Figure 5 shows the measured result. After NIH/3T3 cell seeding (0 h), the measured capacitance values show a gradual increase up to 48 h due to cell growth. After Puromycin injection at 48 h, the capacitance rapidly decreases due to the cell–drug reaction up to 68 h (20 h after Puromycin injection). The reasons for the capacitance changes after Puromycin are as follows: The injected Puromycin disrupts ionic homeostasis in cells, leading to an osmotically induced volume increase and a subsequent capacitance increase [23]. Also, we have checked the possible effect of medium volume change on the measured signals throughout the experiment due to the vaporization, and the effect was negligible. When measured the medium capacitance changes with time, the variation was less than 7% for 96 h. However, the capacitance values with cells were larger than five times compared to the capacitance values without cells (medium only). Thus, the possible effect of medium vaporization during the experiment was negligible. The detailed previous results obtained using reference pattern can be found in [24]. From these results, we have confirmed the stable cell growth and drug reactions in real time using the 0.3 mm reference pattern.

### 3.2. Capacitance Measurements as a Function of Time for Wide and Narrow Impedance Patterns

To check the validity of the wide and narrow gap patterns as impedance biosensors, we have measured capacitance variations with time. For the comparison of the cells, existing on and between the electrodes to capacitance values, we have measured capacitance values as a function of time with various frequencies from 1 kHz to 1 MHz, and 100, 150, 200, and 250 kHz are determined as the adequate frequencies for the capacitance values comparisons both for wide and narrow patterns. The total monitoring time was set as 96 h. The first 48 h were assigned for cell growth monitoring for full growth of the NIH/3T3 cells within the PDMS well, and the second 48 h were for cell–drug reaction monitoring, as we performed in the reference pattern.

**Figure 6:**
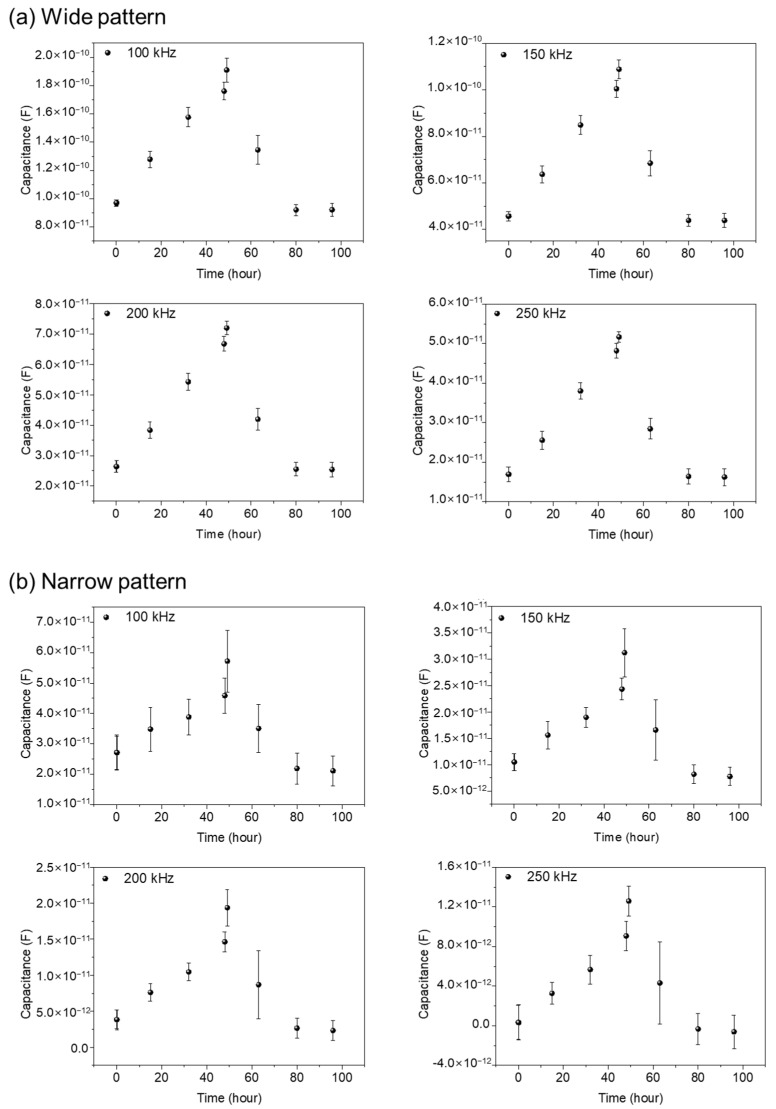
The capacitance variations with time during 96 h both for wide (**a**) and narrow patterns (**b**) for 3 different samples at the frequencies 100, 150, 200, and 250 kHz. The variations in capacitance values are measured at 1, 15, 32, 48, 49, 63, 80, and 96 h, respectively, using three different sensors in the wide gap pattern and the narrow gap pattern, respectively, for comparison and validity of the data. The error bars represent standard deviations obtained from three different experiments for each graph.

Figure 6a,b represents capacitance variations for narrow and wide patterns, respectively. Three different samples are used for data acquisition, both in the wide and narrow patterns. In Figure 6, from the cell seeding time up to 48 h, the measured capacitance values show a gradual increase, reflecting the growth of cells. After Puromycin injection at 48 h, a rapid decrease in capacitance values was monitored, showing the death of the NIH/3T3 cells. A detailed analysis can be found in the previously reported paper [16]. Apparently, the wide and narrow patterns show similar capacitance variations with time. But, the measured capacitance values in the wide pattern show as roughly three to four times larger than those in the narrow pattern, reflecting the importance of the cells existing between the electrodes rather that the cells existing on the electrodes.

### 3.3. Analysis of Capacitance Contribution of the Cells Existing on and between Electrodes

Based on the previous results, cells existing on and between electrodes could be assumed as parallel-connected capacitors in ECIS measurements. Thus, the following equations are possible.

Equations (1) and (2) represent the total capacitance for wide and narrow patterns, respectively. $C_{t}^{w}$ and $C_{t}^{n}$ denote measured total capacitance values of the wide and narrow patterns, respectively.

(1) $$C_{t}^{w}=\frac{A_{e}^{w}}{A_{t}}C_{e}+\frac{A_{g}^{w}}{A_{t}}C_{g}$$

(2) $$C_{t}^{n}=\frac{A_{e}^{n}}{A_{t}}C_{e}+\frac{A_{g}^{n}}{A_{t}}C_{g}$$

$A_{t}$ denotes the total area, including electrode gap and electrode regions, and the total area is 24.51 mm^2^. $A_{e}^{w}$ and $A_{g}^{w}$ denote the electrode and gap area of the wide pattern, and the corresponding areas are 3.42 and 21.09 mm^2^, respectively. $A_{e}^{n}$ and $A_{g}^{n}$ denote the electrode and gap area of the narrow gap pattern within the PDMS inner well, and the corresponding areas are 22.8 mm^2^ and 1.71 mm^2^, respectively. $C_{e}$ and $C_{g}\;$ denote the capacitance of cells existing on electrodes and between electrodes, respectively. By using Equations (1) and (2) and the experimentally acquired values of $C_{t}^{w}$ and $C_{t}^{n}$, we could estimate the cell capacitance values $C_{e}$ and $C_{g}$.

**Figure 7:**
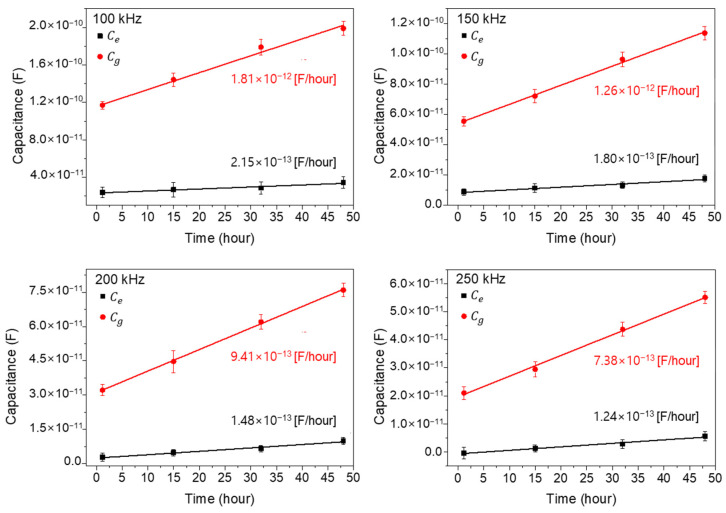
The extracted capacitance values $C_{e}$ and $C_{g}$ for four different times (1, 15, 32, and 48 h: cell growth stage). The closed square and circle represent $C_{e}$ and $C_{g}$, respectively.

Figure 7 shows the extracted capacitance values $C_{e}$ and $C_{g}$ for four different times (1, 15, 32, and 48 h: cell growth stage) by using the aforementioned Equations (1) and (2) and the measured data in Figure 6. In Figure 7, the extracted $C_{g}$ values show as larger than the $C_{e}$ values, by about six times at 1 h, and the differences increase with the increase in cell growth time. Also, the extrapolated slopes, which mean the increasing capacitance value rates with time, are denoted in Figure 7 for each measured frequency. As shown, the $C_{g}$ values increase rapidly compared to the $C_{e}$ values, reflecting the importance of the cells existing between electrodes rather than those existing on electrodes. From this result, it could be concluded that the main cell capacitance values are measured from the cells located between the electrodes rather than on the electrodes. And one more important thing is that the linear increase in capacitance with time confirms the validity of parallel capacitor model both for wide- and narrow-gap patterns [25,26].

**Figure 8:**
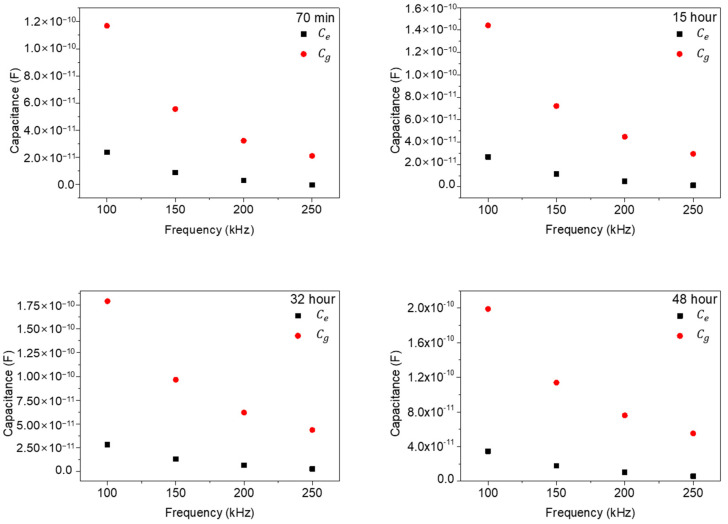
The extracted $C_{e}$ and $C_{g}$ values with frequencies for various times.

In Figure 8, we replotted the results of Figure 7 as a function of frequencies (100, 150, 200, and 250 kHz) to check the frequency dependences of the $C_{e}$ and $C_{g}$ values. As shown in Figure 8, the extracted $C_{e}$ and $C_{g}$ values rapidly decrease with the increase in measured frequency and gradually approach a zero value, which signifies a general frequency dependence in impedance measurements. However, the differences between $C_{e}$ and $C_{g}$ also rapidly decrease with the increase in frequency. This behavior represents the strong low frequency dependency characteristics of NIH/3T3 cell. In other words, in ECIS measurements of NIH/3T3 cells, low frequency ranges around 100 kHz could give improved signal characteristics.

**Figure 9:**
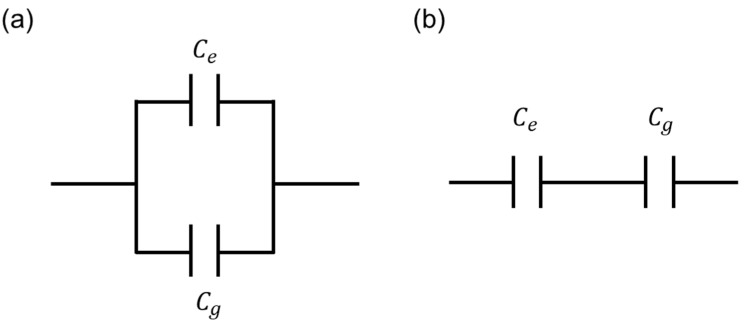
Equivalent circuit model in case of parallel (**a**) and serial connections (**b**), respectively, for $C_{e}$ and $C_{g}$. From Equation (1) and Figure 7 and Figure 8, the proper equivalent circuit model is the (**a**) parallel connection model.

Figure 9 shows the two possible equivalent circuit models between $C_{e}$ and $C_{g}$ which are introduced for the clear summary of this work. As already discussed in Equation (1), the parallel connection model (Figure 9a) is proper in this work. From Figure 6 and Figure 7, the extracted $C_{e}$ and $C_{g}$ values showed good coincidences between experimental data with parallel model-based extracted data, which are extracted by using Equation (1). Finally, from Equation (1) and Figure 7 and Figure 8, the proper equivalent circuit model is the parallel connection model. For the cell capacitance measurement equivalent model, considering the cell resistance, cell capacitance, and medium resistance, the detailed model and analysis are discussed in the previously reported work [27]. 

In summary, the main cell capacitance values are measured from the cells existing between the electrodes rather than on the electrodes, and the parallel capacitance model is proper in the ECIS measurement. Finally, the minimized narrow electrodes with maximized electrode gap distance structures are recommended for the accurate measurement of cell capacitance variations in the ECIS method.

## 4. Conclusions

This study aimed to investigate the capacitance dependence of NIH/3T3 cells with the existing locations either on the electrodes or between the electrodes of an impedance biosensor. For the comparison, two different patterns were fabricated: a wide gap pattern with an electrode gap of 3.7 mm and a narrow gap pattern with an electrode gap of 0.3 mm. The capacitance values were measured with the cell growth, and a detailed analysis was performed with the obtained capacitance values. The results showed that the parallel-connected capacitors model was proper, with the cells both existing on the electrodes and between the electrodes with the cell population increase. Moreover, from the detailed analysis, the overall contributions of the cells on the electrodes compared to the cells existing between electrodes were less than 10%, showing an importance in the cells between the electrodes. Thus, in the designing of ECIS impedance biosensors, a maximum gap spacing with a minimized electrode area is essential for effective signal acquirement.

## Data Availability

The data that support the findings of this study are available upon request from the authors.
